# Subtask Segmentation of Timed Up and Go Test for Mobility Assessment of Perioperative Total Knee Arthroplasty †

**DOI:** 10.3390/s20216302

**Published:** 2020-11-05

**Authors:** Chia-Yeh Hsieh, Hsiang-Yun Huang, Kai-Chun Liu, Kun-Hui Chen, Steen Jun-Ping Hsu, Chia-Tai Chan

**Affiliations:** 1 Department of Biomedical Engineering, National Yang-Ming University, Taipei 11221, Taiwan; g39904006@ym.edu.tw (C.-Y.H.); huangshoy@ym.edu.tw (H.-Y.H.); 2 Research Center for Information Technology Innovation, Academia Sinica, Taipei 11529, Taiwan; t22302856@citi.sinica.edu.tw; 3 Department of Orthopedic Surgery, Taichung Veterans General Hospital, Taichung 40705, Taiwan; orthochen@gmail.com; 4 Department of Biomedical Engineering, Hungkuang University, Taichung 43302, Taiwan; 5 Department of Information Management, Minghsin University of Science and Technology, Hsinchu 30401, Taiwan; steenhsu@must.edu.tw

**Keywords:** subtask segmentation, timed up and go (TUG) test, wearable sensor, perioperative total knee arthroplasty

## Abstract

Total knee arthroplasty (TKA) is one of the most common treatments for people with severe knee osteoarthritis (OA). The accuracy of outcome measurements and quantitative assessments for perioperative TKA is an important issue in clinical practice. Timed up and go (TUG) tests have been validated to measure basic mobility and balance capabilities. A TUG test contains a series of subtasks, including sit-to-stand, walking-out, turning, walking-in, turning around, and stand-to-sit tasks. Detailed information about subtasks is essential to aid clinical professionals and physiotherapists in making assessment decisions. The main objective of this study is to design and develop a subtask segmentation approach using machine-learning models and knowledge-based postprocessing during the TUG test for perioperative TKA. The experiment recruited 26 patients with severe knee OA (11 patients with bilateral TKA planned and 15 patients with unilateral TKA planned). A series of signal-processing mechanisms and pattern recognition approaches involving machine learning-based multi-classifiers, fragmentation modification and subtask inference are designed and developed to tackle technical challenges in typical classification algorithms, including motion variability, fragmentation and ambiguity. The experimental results reveal that the accuracy of the proposed subtask segmentation approach using the AdaBoost technique with a window size of 128 samples is 92%, which is an improvement of at least 15% compared to that of the typical subtask segmentation approach using machine-learning models only.

## 1. Introduction

Osteoarthritis (OA) is a major source of disability that can affect the activities of daily living (ADLs) and the quality of life of elderly individuals. The incidence of knee OA is increasing because of increased age and life expectancy, increased obesity and lack of physical activity. A previous study showed that more than 250 million people worldwide suffer from knee OA [1]. Total knee arthroplasty (TKA) is one of the most common treatments for people with severe knee OA, which is in the terminal stage of knee OA [2,3]. TKA can reliably relieve pain and improve mobility function and health-related quality of life [4]. To assist clinical evaluation and assessment, the measurement of the progress is important for perioperative TKA. Measurements and assessments can be used to inform clinical professionals, patients, and caregivers about therapeutic efforts. Furthermore, outcomes from measurements and assessments may improve healthcare resources and service management.

Currently, patient-based and performance-based measures are common methodologies to measure the mobility status of perioperative TKA recovery progress. Patient-based measures are generally executed by clinical professionals, who use mail-in questionnaires, telephone interviews, and screening tools to investigate the health-related status of patients for perioperative TKA. However, patient-based measures present some issues. The first is that most of these measures rely on forced choice queries or subjective statements presented by the patients or primary caregivers. Such information may be inaccurate or have bias because it relies on the recall of events or a snapshot observation of function. The second is individual variance between the different raters. The raters provide subjective comments for patient performance on patient-based measures.

**Figure 1:**
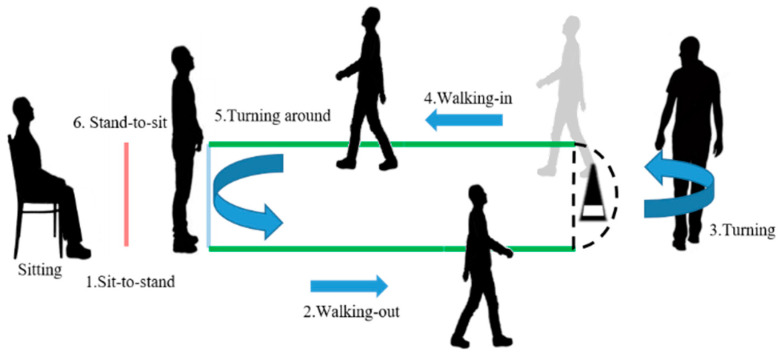
The complete timed up and go (TUG) test.

Performance-based measures focus on the measurement and assessment of physical functions by capturing the ability of patients to perform a unitary task or multiple tasks. Various measures of unitary tasks have been proposed for mobility assessment, such as the chair stand test (CST), six-minute walk test (6MWT) and stair climb test (SCT) [5]. These measures are often assessed by counting repetition, timing, range of motion (ROM), and total elapsed time. However, some important clinical fine-grained information might be neglected while limiting the measurement of specific information from a unitary task. A test with multiple tasks can measure the fine-grained and general information of physical functions. The timed up and go (TUG) test consisting of multiple tasks is widely utilized to measure basic mobility and balance capabilities in patients who are elderly and frail elderly, and patients suffering from Parkinson’s disease (PD), multiple sclerosis (MS), stroke, lumbar degenerative disc disease, lower limb amputations and chronic obstructive pulmonary disease (COPD) [6,7,8,9,10,11,12,13,14]. Some studies measured the total time execution of the TUG test. However, the TUG test as shown in Figure 1 contains various subtasks, such as sit-to-stand, stand-to-sit, walking, and turning tasks. By extracting the information from each subtask of the TUG test, the fine-grained outcomes can reveal important clinical information related to subjects. The information, such as elapsed time, balance and ROM of each subtask, can be used to assist professionals in clinical interventions and distinguish the functional recovery of patients.

The conventional measurement of a TUG test is manual execution by a rater who uses a stopwatch to record the total elapsed time of the TUG test. Manual execution may suffer from the issues of subjectiveness and rater biases. To tackle these issues, subtask segmentation and identification of TUG tests using inertial measurement units (IMUs) have been proposed to obtain fine-grained TUG information and gait characteristics objectively and reliably in clinical environments [15,16,17,18,19]. However, there are still several technical challenges in the current approaches for perioperative TKA. One of the most important challenges is the motion variability in different recovery progresses and individuals. Due to personal perception and recovery status, motion characteristics are diverse among different stages of perioperative TKA [20].

**Figure 2:**
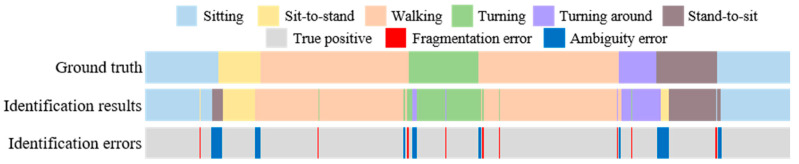
Fragmentation and ambiguity errors in the conventional subtask identification approach.

Fragmentation is another challenge in the subtask segmentation because of the unavoidable misclassification during the identification processes. This misclassification often leads a complete segment to be divided into fragments, especially during the motion transitions. The last challenge is ambiguity, which often occurs while there are highly overlapping motion characteristics between the defined activities, such as walking-out and walking-in, and turning and turning around in the TUG test. The issue of ambiguity may decrease the recognition ability of the subtask identification approaches. Figure 2 shows the fragmentation and ambiguity errors in subtask segmentation and identification.

In this work, a novel subtask segmentation approach using machine-learning and knowledge-based postprocessing approaches is proposed to tackle the technical challenges of classifying the subtasks of the TUG test—i.e., motion variability, fragmentation and ambiguity. A series of signal-processing mechanisms and pattern recognition approaches involving machine learning-based multi-classifiers, fragmentation modification and subtask inference are designed and developed to recognize the subtasks of the TUG test on TKA patients. The main contributions are as follows:

- The proposed subtask segmentation approach, including machine learning-based multi-classifiers, fragmentation modification and subtask inference, can effectively improve the segmentation performance of the TUG test.
- The reliability and effectiveness of the proposed approach is validated on 26 TKA patients and four phases of the perioperative TKA, including preoperative, postoperative, postoperative 2-week and postoperative 6-week.
- The experimental results reveal that the accuracy of the proposed subtask segmentation approach for the TUG test is 92%, which is an improvement of at least 15% compared to that of the typical subtask segmentation approach using machine-learning models only.

## 2. Related Work

### 2.1. Windowing Segmentation Technique

Windowing segmentation techniques process the sensing signal stream into smaller data segments. Various techniques have been proposed for signal segmentation, including sliding window, event-defined and activity-defined techniques [21,22]. First, the sliding window technique, the most widely employed technique in activity recognition, can divide the signal stream into a sequence of discrete segments. The advantages of the sliding window technique are the ease of implementation and the reduction in computation complexity. In the feature extraction process of machine learning and deep learning, the sliding window technique has been widely utilized to segment data streams.

Second, an event-defined technique can divide the continuous signal stream into segments by event detection. The detected events are mainly used for recognizing the occurrence time of the interesting activity. For example, detections of the heel-strike and heel-off motions are generally utilized for the identification of the complete gait cycle. Sant’Anna and Wickström proposed a symbol-based approach to detect the phases of gait and convey information from accelerometer signals [23].

The last technique, the activity-defined technique, locates the initial and ending time of an activity based on detecting the changes in activity. Yoshizawa et al. proposed an automatic segmentation technique that is based on a spectral transition measure to detect the changing point of activity from sensor data [24]. The changing point of activity is defined by the change in activity from static to dynamic activity and vice versa. Masaki et al. utilized the discrete wavelet transform to detect changing frequencies in walking patterns [25]. Ngo et al. proposed an approach to segment the similar gait action, such as walking on flat ground, up/downstairs, and up/down a slope. The scale-space technique, orientation-compensative matching algorithm, and incorporation of the interclass relationship in the feature vector are utilized and carried out to accurately classify similar actions [26]. Apart from these segmentation approaches, the motions can also be identified through external identification techniques, such as rule-based algorithms and machine learning-based classifiers.

### 2.2. Identification Technique

The sensing signal stream can be divided into many data segments through the segmentation technique; then, the data segments should be identified as motions by the identification technique. Identification techniques are primarily classified into two types: rule-based and machine learning-based (ML-based) identification techniques. First, the rule-based identification technique uses rules to detect the starting or transition point of motion following physical attributes. As an example, the motion of sit-to-stand was divided into four phases, namely the flexion momentum, momentum transfer, extension and stabilization phases [27]. By detecting the flexion momentum and stabilization phase, the sit-to-stand motion can be identified. During the first flexion momentum phase, the trunk and pelvis rotate anteriorly, and the thighs, shanks and feet remain stationary. The stabilization phase begins after the hip extension velocity reaches approximately zero until motion stabilization. Therefore, the actions of the trunk bending forward and the hip extension stopping are regarded as the first and last physical attributes of the sit-to-stand motion, respectively. Nguyen et al. [16] developed an automated recognition and segmentation algorithm to identify motor activity patterns in daily living tasks. The proposed algorithm defined the transition point as the beginning and ending of each activity following the biomechanics of movement.

The ML-based identification technique constructs a model using features from training data to predict or solve the given problem. Commonly used machine-learning techniques include naïve Bayesian (NB), support vector machine (SVM), k-nearest neighbor (kNN), random forest (RF), and decision tree (DT) methods [22,28,29]. Nam and Park [30] proposed a machine learning-based approach to recognize 11 daily physical activities of children using a diaper-worn device consisting of an accelerometer and a barometric air pressure sensor. The machine learning-based techniques utilized in the approach included the NB, Bayes net (BN), SVM, kNN, decision table, DT, multi-layer perceptron (MLP), and logistic regression techniques. Chelli and Pätzold [31] developed a machine-learning framework using acceleration and angular velocity data from two public databases for fall detection and daily living activity recognition. The machine-learning framework included an artificial neural network (ANN), kNN, SVM with a quadratic kernel function, and ensemble bagged tree (EBT). Reinfelder et al. [17] proposed an automated TUG phase classification algorithm using four machine learning-based techniques and validated it with 16 Parkinson’s disease patients. Two IMUs were attached on the lateral side of both shoes to record the acceleration and angular velocity during performing the TUG test. The results showed that the best average sensitivity was 81.8% using the SVM technique. Hellmers et al. [32] proposed an IMU-based hierarchical classification model to classify activities during the TUG test. The first layer distinguished the activity states (static, dynamic, and transition) and the second layer classified the possible activities of each state. The overall F1-score results achieved were over 70%. In addition, the best F1-score results achieved 97% for dynamic activity walking.

## 3. Methods

### 3.1. Subjects and the TUG Test Protocol

The experiment recruited 26 patients with severe knee OA (5 males and 21 females; 69.15 ± 6.71 years old; height: 154.65 ± 8.26 cm; weight: 65.50 ± 9.83 kg), consisting of 11 patients with bilateral TKA planned, and 15 patients with unilateral TKA planned. The TUG test protocol was performed in four phases for perioperative TKA, namely, preoperative, postoperative, postoperative 2-week and postoperative 6-week. In each phase, the patient performed the 5- and 10-m TUG tests three times. Each TUG test was defined as one TUG trial. To perform a complete TUG test, the patient was instructed to sit for 5 s on a chair, stand up from the chair, walk 5/10 m, turn around a cone on the ground, walk back to the chair, sit down on the chair and remain sitting for 5 s. The TUG test was divided into six lower limb subtasks: sit-to-stand, walking-out, turning, walking-in, turning around and stand-to-sit. An illustration of the complete TUG test is shown in Figure 1. Patients can perform the TUG test with a walker for safety in the experiment.

To provide the ground truth for the proposed subtask segmentation approach, a camera-based system utilized two cameras, which were synchronized with inertial sensor nodes, to provide reference data for manual labeling. The sampling rate of the cameras is 30 Hz. One camera was placed behind the participant to capture the back view, and the other camera was placed on the left side of the participant to capture the side view. The researcher labeled the timestamp of ground truth manually based on the recorded video. The information of subtasks during the TUG test was determined by manual labeling, which includes the initial and ending points of each subtask.

### 3.2. The Proposed System

**Figure 3:**
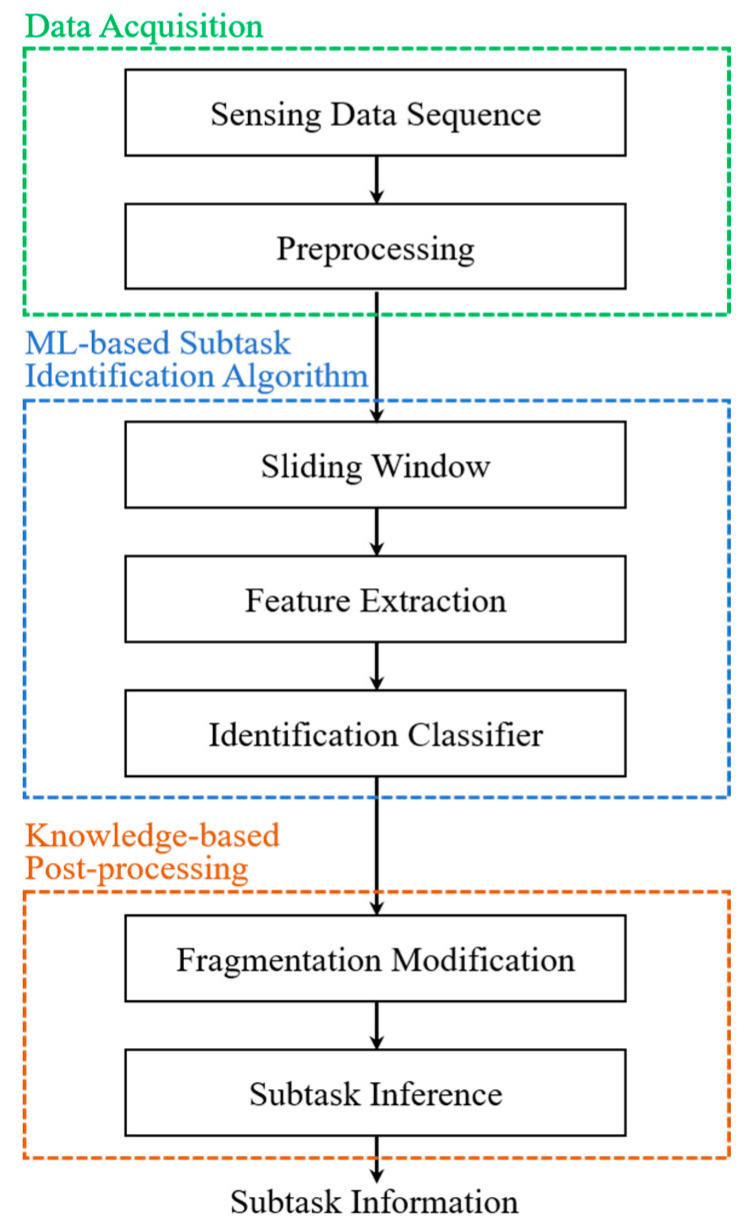
The functional diagram of the subtask identification system.

**Figure 4:**
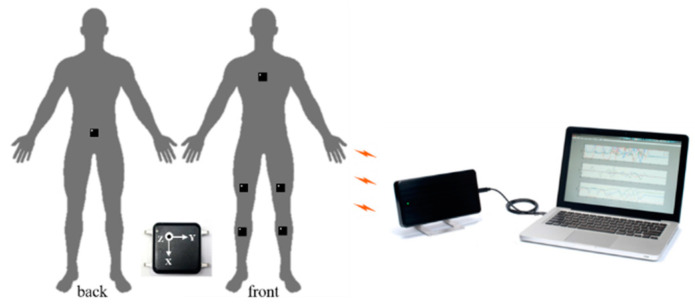
Schematic view of the sensor attachment on the participants and the wireless transfer of the laptop.

The functional diagram of the proposed subtask segmentation approach is shown in Figure 3. There are three stages in the proposed system, including data acquisition, ML-based subtask identification algorithm, and knowledge-based postprocessing. To start with the data acquisition, including the sensing data sequence and preprocessing steps, six research-grade inertial sensor nodes (OPAL, APDM, Inc., Portland, OR, USA), each of which contained a triaxial accelerometer, gyroscope, and magnetometer, were utilized. In this study, only acceleration collected by the triaxial accelerometer and angular velocity collected by the triaxial gyroscope were acquired. Six sensor nodes were placed on the chest, lower back, right thigh, left thigh, right shank, and left shank of each subject. The sensor placement for chest and lower back was around the fourth rib and fifth lumbar vertebra, respectively. The sensor placement for the thigh and shank was about 15 cm above and 13 cm below the center of the knee joint, respectively. The sensing data were recorded at a 128-Hz fixed sampling rate and transferred wirelessly to a laptop for storage. A schematic view of the sensor attachment on the participants and the wireless transfer of the laptop are shown in Figure 4.

In the preprocessing step, the moving average filter was first adopted to remove random noise, which arises from various sources, such as unexpected individual movement and muscle vibration during the TUG test. Then, the resultant acceleration ($A_{T}$) and resultant angular velocity ($G_{T}$) were calculated by Equations (1) and (2), where $a_{x}$, $a_{y}$, and $a_{z}$ are the accelerations in the *x*-, *y*-, and *z*-axes, respectively, and $\omega_{x}$, $\omega_{y}$, and $\omega_{z}$ are the angular velocities in the *x*-, *y*-, and *z*-axes, respectively.

(1) $$A_{T}=\sqrt{a_{x}{}^{2}+a_{y}{}^{2}+a_{z}{}^{2}}$$

(2) $$G_{T}=\sqrt{\omega_{x}{}^{2}+\omega_{y}{}^{2}+\omega_{z}{}^{2}}$$

### 3.3. ML-Based LLM Identification Algorithm

The ML-based subtask identification algorithm has three steps, including sliding window, feature extraction and identification classifier. First, the sensing data were partitioned into segments by different window sizes with a 50% overlap in the sliding window step. Since the elapsed time of each motion is unbalanced in the TUG test, there may be a tradeoff between the performance and window size selection. In this study, the elapsed time of sit-to-stand/stand-to-sit and one step in the walking condition was about one second. Therefore, 96, 128 and 160 samples (0.75, 1, 1.25 s) were adopted as window sizes. Then, 8 types of time domain statistical features were extracted from the segments, including the mean, standard deviation, variance, maximum, minimum, range, kurtosis, and skewness. The extracted features are listed in Table 1. There were a total of 384 features (8 types × 8 axes × 6 sensor nodes) extracted for the machine-learning training classifier.

Five machine-learning techniques were adopted as the identification classifier, and their performances in subtask identification are compared.

1. Support Vector Machine (SVM)

An SVM is a supervised machine-learning technique originally used for binary classification by constructing the optimal separating hyperplane and margins between two datasets. There are different types of kernel functions for an SVM classifier, such as linear, polynomial, Gaussian, sigmoid, and radial basis functions (RBFs). Considering computational complexity, the multi-class SVM classifier with the linear kernel function is applied for subtask identification.

2. K-Nearest Neighbor (kNN)

KNN is a simple algorithm that stores all training data and classifies testing data based on distance functions. The parameter k, which is a positive integer, is defined as the number of the nearest neighbors of the testing data. The class of the testing data is decided by a vote of these k neighbors. Because k is sensitive to data distribution, it commonly uses a conventional constant limited among 1, 3, 5, 7, and $\sqrt{n}$, where n is the number of training data [33]. In this study, the parameter k was tested in the range between 1 and 21, and the best results were obtained by a value of 9.

3. Naïve Bayesian (NB)

The NB classifier is a probabilistic model based on the Bayes theorem of probability. This model considers each feature to contribute independently to the probability of each class, and then the posterior probability for each class is calculated. The testing data are classified into particular classes by the maximum posteriori probability.

4. Decision Tree (DT)

A DT classifier is built from the set of rules based on features of the training data. The testing data follow the rules to fall under one class. Classification and regression trees (CARTs) are implemented to train subtask identification in this study.

5. Adaptive Boosting (AdaBoost)

Boosting is an ensemble machine-learning algorithm that aims to create a strong classifier by repeatedly building weak classifiers. AdaBoost combines multiple weak classifiers with a weighted sum to construct a stronger classifier. The weak classifiers adapted in AdaBoost are DTs. Each training datum is allotted a weighting to train a weak classifier. The training data misclassified by the previous weak classifier obtain a higher weighting in the next weak classifier. In contrast, the training data correctly classified by the previous weak classifier obtain a lower weighting in the next weak classifier.

### 3.4. Knowledge-Based Postprocessing

**Figure 5:**
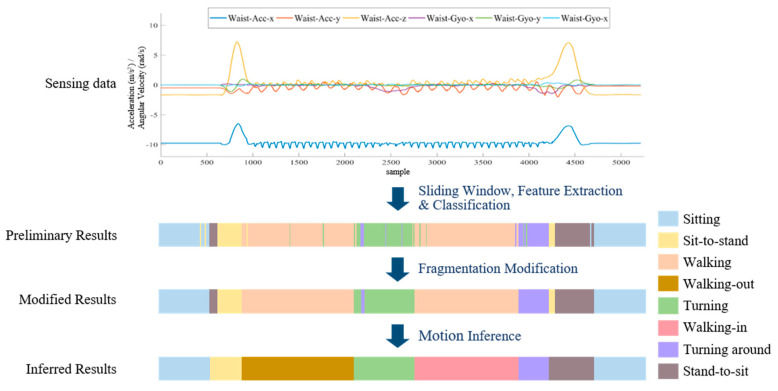
An example of the modified and inferred results.

The knowledge-based postprocessing stage includes fragmentation modification and subtask inference steps, which aim to modify the misclassification results from the ML-based subtask identification algorithm. An example of a knowledge-based postprocessing result is illustrated in Figure 5. The fragmentation modification step is utilized to modify the misidentified segments firstly.

The pseudocode of the fragmentation modification algorithm is delineated in Algorithm 1. An identified segment sequence is defined as $TUG=\left\{(subt_{i}|i=1,2,3,\ldots ,N\right\}$, where $subt_{i}$ is the *i*th subtask segment, 1≤i≤N, and *N* is the total number of identified segments. The modified segment sequence is defined as $MTUG=\left\{(msubt_{i}|i=1,2,3,\ldots ,N\right\}$, where $msubt_{i}$ is the *i*th modified subtask segment, 1≤i≤N, and *N* is the total number of identified segments. Each subtask ($subt_{i}$) and modified subtask ($msubt_{i}$) belong to SSUBT, and SSUBT is the set of distinct semantic subtasks $SSUBT=\;\left(\left(ssubt_{j}\right)|1\leq j\leq k\right)$, where k is the number of defined semantic subtasks. In this study, seven semantic subtasks are defined—(k = 7) {“Sititng”, “Sit−to−stand”, “Walking−out”, “Turning”, “Walking−in”, “Turning around”,  “Stand−to−sit”}.

**Figure 6:**
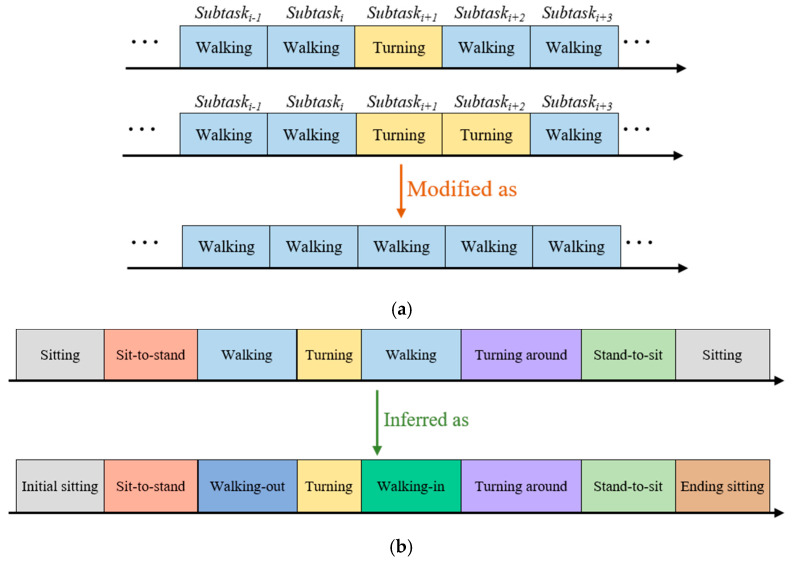
The knowledge-based postprocessing: (**a**) the fragmentation modification and (**b**) the motion inference.

At the beginning of the fragmentation modification process, the first segment and the last two segments are indicated to sitting, referring to line 1 to line 3 and line 12 to line 14 in the pseudocode of the fragmentation modification algorithm. This is because the starting and ending subtask in the TUG test is sitting. Next, the subtasks of one or two continuous segments differing from those of forward and backward segments are regarded as misidentified segments. The subtask of misclassified segment(s) would be modified as the subtask of the forward and backward segments with reference to line 4 to line 9 of the fragmentation modification algorithm. An illustration of the fragmentation modification is shown in Figure 6a.
**Algorithm** ** 1:** Fragmentation modification algorithm in the knowledge-based postprocessing stage**Input:**An identified segments sequence $\;TUG=\left\{(subt_{i}|i=1,2,3,\ldots ,N\right\}$, The *i*th subtask segment $subt_{i}$**Output:**A modified and identified segments sequence $MTUG=\left\{(msubt_{i}|i=1,2,3,\ldots ,N\right\}$, The *i*th modified subtask segment $msubt_{i}$1:$subt_{1}=ssubt_{1}$//$ssbt_{1}$ is the semantic subtask of sitting.2:$subt_{N-1}=ssubt_{1}$3:$subt_{N}=ssubt_{1}$4:**for**i from 2 to N−2
**do**5: **if**
$subt_{i}$ != $subt_{i-1}$ && $subt_{i-1}$ == $subt_{i+1}$
**then**6:  $subt_{i}$ = $subt_{i-1}$7: **else if**
$subt_{i}$ != $subt_{i-1}$ && $subt_{i+1}$ != $subt_{i-1}$ && $subt_{i+2}$ == $subt_{i-1}$
**then**8:  $subt_{i}$ = $subt_{i-1}$9: **end if**10:$msubt_{i}$ = $subt_{i}$11:**end for**12:$msubt_{1}=ssubt_{1}$13:$msubt_{N-1}=ssubt_{1}$14:$msubt_{N}=ssubt_{1}$15:**return**MTUG

**Figure 7:**
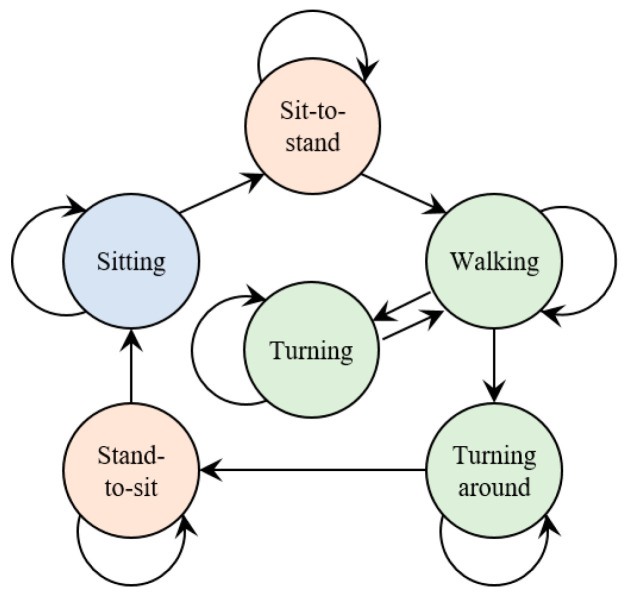
The subtask state transition diagram.

Second, the subtask inference step considers the certain temporal order of the subtasks during the TUG test. The defined temporal order of occurring subtasks is initial sitting, sit-to-stand, walking-out, turning, walking-in. turning around, stand-to-sit, and ending sitting. This scenario is illustrated by the state transition diagram in Figure 7. The subtask state transition diagram includes six states: sitting, sit-to-stand, walking, turning, turning around, and stand-to-sit. The initial posture and ending posture during the TUG test are defined as subtasks of initial sitting and ending sitting. The temporal order of walking-in and walking-out subtasks is inferred by the turning subtask. While the temporal order is confirmed, the subtask segments can be gathered. Finally, the subtasks are identified by the proposed algorithm. The subtask identification approach classifies subtasks into 8 classes, namely, initial sitting, sit-to-stand, walking-out, turning, walking-in, turning around, stand-to-sit, and ending sitting. The subtask information can be obtained, including the initial boundary, ending boundary, and duration of subtask. An illustration of the subtask inference is shown in Figure 6b.

### 3.5. Evaluation Methodology

The p-fold cross validation approach was employed to evaluate the segmentation and identification performance, where *p* = 5. The approach randomly divides all collected trials into 5 partitions, 4 partitions as the training set and 1 partition as the testing set, and iterates 5 times until each partition is used as the testing set. The proposed system performance is analyzed with three evaluation metrics, namely, sensitivity, precision, and accuracy. These metrics are computed by Equations (3)–(5), where true positive, false positive, true negative, and false negative are indicated by TP, FP, TN, and FN, respectively.

(3) $$Sensitivity=\frac{TP}{TP+FN}$$

(4) $$Sensitivity=\frac{TP}{TP+FN}$$

(5) $$Accuracy=\frac{TP+TN}{TP+FP+TN+FN}$$

**Figure 8:**
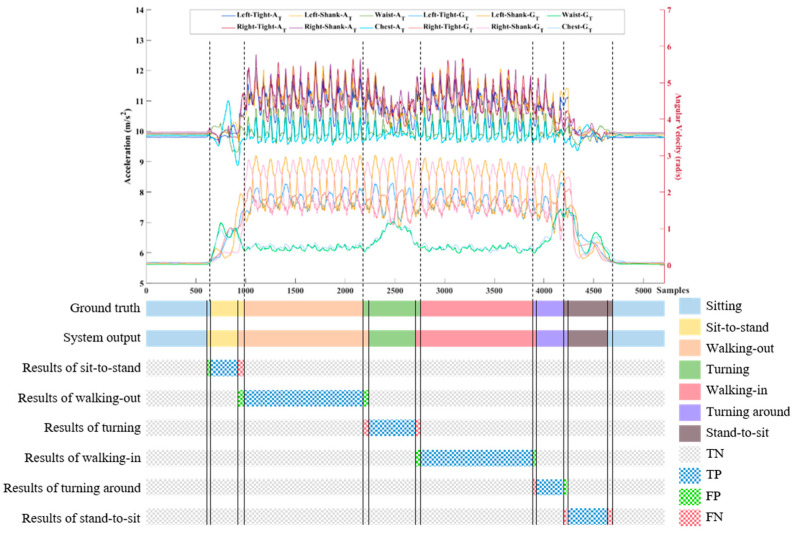
An example of the 10-m TUG test in subtask identification. Only the triaxial acceleration and angular velocity of the waist are plotted in the example. The system output is compared against to the ground truth in terms of true negative (TN), true positive (TP), false positive (FP), and false negative (FN).

The sensitivity and precision are represented for the evaluation of each subtask segmentation. However, accuracy in the multi-class classifier is calculated as the average accuracy for evaluation of the subtask segmentation approach. The definition of the true positive, false positive, true negative and false negative performance metrics in the continuous data stream is shown in Figure 8, using a 10-m TUG test as an example. Only the triaxial acceleration and angular velocity of the waist are plotted in the example. The system output is compared against the ground truth in terms of TN, TP, FP, and FN.

## 4. Results

There were 624 trials (26 patients × 6 TUG trials × 4 phases) of collected TUG tests, including 312 TUG trials of 5 m and 312 TUG trials of 10 m. Since the experimental protocol includes four phases in the perioperative TKA, two methodologies (multi-classifier and single classifier) were utilized to preliminarily train the subtask identification classifier. In the multi-classifier, each phase trained one identification classifier independently. In other words, a total of four classifiers were trained for subtask identification. In the single classifier, one classifier was trained from data of all four phases. The performances of the multi-classifier and the single classifier are presented in Table 2.

**Figure 9:**
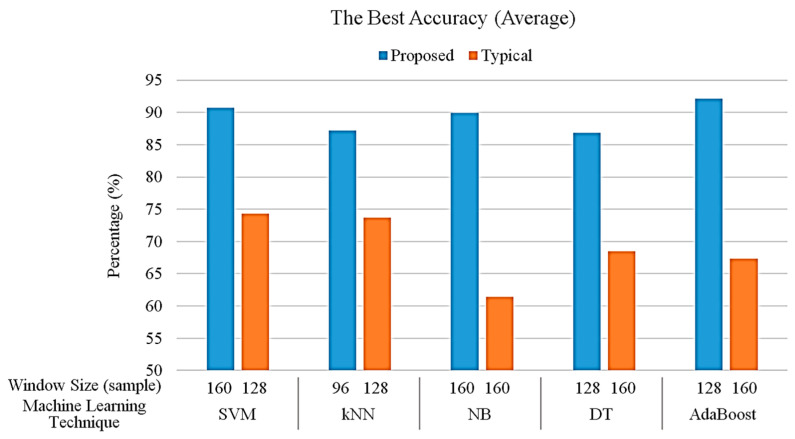
The best overall accuracy in each technique for proposed and typical machine-learning algorithms.

The best overall accuracy of each machine-learning algorithm in the proposed and typical machine-learning algorithm (without knowledge-based postprocessing) are shown in Figure 9. The proposed algorithm using the AdaBoost technique with a window size of 128 samples achieved the best overall accuracy of 92.14%. Compared to the proposed algorithm, the best overall accuracy of the SVM, kNN, NB, DT and AdaBoost techniques in the typical machine-learning algorithm achieves only 74.33% with a window size of 128 samples, 73.73% with a window size of 128 samples, 61.45% with a window size of 160 samples, 68.45% with a window size of 160 samples and 67.38% with a window size of 160 samples, respectively. Generally, the overall performance of the proposed algorithm outperforms the typical algorithm regardless of the machine-learning technique. The performance results of each phase in the proposed algorithm with different window sizes are shown in Table 3 in detail.

The performances in the preoperative phase are higher than the performances in other phases over each window size. The highest accuracy in the preoperative, postoperative, postoperative 2-week, and postoperative 6-week phases occurred using AdaBoost with a window size of 96 samples, AdaBoost with 128 samples, DT with 96 samples, and SVM with 96 samples, respectively. The highest accuracy in the window sizes of 96, 128, and 160 samples occurred when using AdaBoost in the preoperative phase and the postoperative phase. At first glance, the subtask identification approach using the AdaBoost and SVM techniques outperforms those using kNN, NB and DT techniques. The highest accuracy in all adopted techniques occurs in the preoperative phase with a window size of 96 samples. Meanwhile, the performances of each subtask using AdaBoost with a window size of 96 samples (preoperative), AdaBoost with 128 samples (postoperative), DT with 96 samples (postoperative 2-week), and SVM with 96 samples (postoperative 6-week) are shown in Table 4. Generally, the sensitivity of walking-out and walking-in outperforms the sensitivity of other subtasks over the phases. The sensitivity of sit-to-stand and stand-to-sit subtasks is worse than that of others. Furthermore, the turning around subtask underperforms in terms of precision compared to others.

## 5. Discussion

In a clinic environment, the information of the TUG test is traditionally obtained through manual execution by a rater using a stopwatch to measure the total elapsed time or the elapsed time of each subtask. Manual execution may involve issues of intrarater and inter-rater bias because of the repeating measurement by identical or different raters. The biases have a great negative effect on measuring several short-term activities—e.g., sit-to-stand and stand-to-sit. To measure objectively and reduce manual biases, the main objective of this study is to design a TUG subtask segmentation approach using machine-learning models and knowledge-based approaches to identify the subtasks during the TUG test for perioperative TKA. The performance of the typical algorithm in overall accuracy ranges from 60% to 74% with respect to different techniques and window sizes. Compared to the performance of the typical algorithm, the best overall accuracy of the proposed algorithm can achieve 92% using AdaBoost with a window size of 128 samples. Obviously, the proposed approach can effectively tackle the technical challenges in subtask identification, including variability, fragmentation and ambiguity.

Based on Table 3, the proposed algorithm using SVM and AdaBoost techniques have better overall accuracy than those using other techniques and the proposed algorithm using kNN underperforms compared to those using others. Therefore, SVM and AdaBoost techniques are recommended to identify the subtasks in each phase of perioperative TKA, especially in the postoperative phase. Additionally, there are sizable differences between the performances using different machine-learning techniques in the postoperative phase. This is because great subtask variability and individual differences exist in the postoperative phase, caused by postoperative pain and adaptation period from a new implant after TKA surgery. The experimental results show that the selection of a machine-learning technique in the postoperative phase has a great impact on the algorithm performance.

According to Table 4, the sensitivity of walking-out and walking-in presents a better performance than that of the other subtasks in each phase, and that of sit-to-stand and stand-to-sit generally underperform compared to the other subtasks. This is because the elapsed time of each subtask is unbalanced in the TUG test, which results in a disproportionate ratio of identification error in each subtask. In the situation of identical misidentified data points, a subtask with a shorter elapsed time has greater influence on the identification error than one with a longer elapsed time. Moreover, the indistinct boundary between subtasks often confuses the proposed segmentation approach. The patient may execute the next subtask while he/she is still performing the current subtask. For example, a patient may start walking ahead before he/she finishes sit-to-stand, or a patient may start stand-to-sit while turning around.

**Figure 10:**
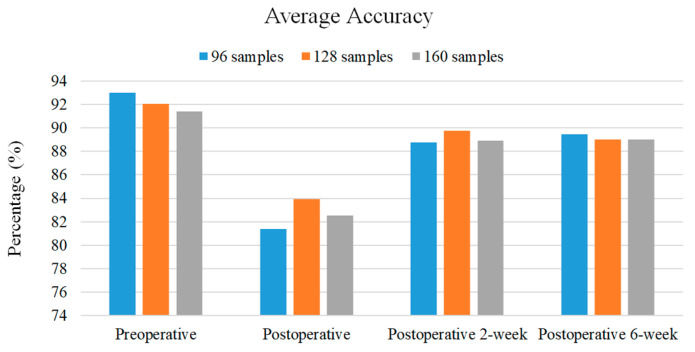
The average accuracy of proposed algorithm with different window sizes in each phase.

The window size of the sliding window is a common issue in activity recognition and motion identification. A large window size may blur the pattern of target motion due to the inclusion of patterns of other motions. However, a small window size cannot cover the entire pattern of target motion, leading to a training error. The use of a suitable window size has the potential to increase the performance. Since the elapsed time of each subtask is unbalanced in the TUG test, there may be a tradeoff between the performance and window size selection. This study adopted 96, 128 and 160 samples (0.75, 1 and 1.25 s) as the window size. The average accuracy of different adopted window sizes over each phase is shown in Figure 10. Generally, the most suitable window size is 96 samples for the preoperative and postoperative 6-week phases and 128 samples for the postoperative and postoperative 2-week phases. Nevertheless, the differences between the average accuracy using different window sizes are within 3%. By the proposed approach, all adopted window sizes can be utilized as a suitable window size.

The number of sensors implicating collected data volume may impact on recognition ability and computational complexity. Based on the previous works [34,35,36], the performance of activity recognition using more sensors is higher than that using only one sensor. The reason is that more movement patterns can be embodied in the recognition models while using more sensors. To involve more movement patterns from various body parts, six sensors were utilized to collect the movement data in this study. In the future, the segmentation performance using a different number of sensors will be evaluated and the best combination of sensor placement will be investigated.

In this study, the perioperative TKA was divided into four phases to collect sensing data for the TUG test: preoperative, postoperative, postoperative 2-week and postoperative 6-week. Therefore, four classifiers were trained for four phases because of differences in the recovery progress between phases. In addition, the patients were allowed to perform the TUG tests with a walker for safety in the experiment. The subtask pattern of sensing data from the patient performing the TUG test with a walker is different from that without a walker. In the experiment, all patients performed the TUG test without a walker in the preoperative phase and with a walker in the postoperative phase. In the postoperative 2-week and postoperative 6-week phases, the walker is an optional auxiliary appliance when patients perform the TUG test. Therefore, we plan to train two classifiers to separate the data of patients performing the TUG test with and without a walker in the future.

## 6. Conclusions

The outcome measurement and quantitative assessment are important processes for perioperative TKA. The TUG test has been realized and used to validate tools to measure basic mobility and balance capabilities. The outcome of subtasks can reveal important clinical information related to subjects. This study aims to design a subtask segmentation approach using machine-learning models and knowledge-based postprocessing during the TUG test for perioperative TKA. A series of signal-processing mechanism and pattern recognition approaches involving machine learning-based multi-classifiers, fragmentation modification and subtask inference are designed and developed to tackle technical challenges—i.e., motion variability, fragmentation and ambiguity. The reliability of the proposed approach was validated with 26 TKA patients. The experimental results reveal that the accuracy of the proposed subtask segmentation approach using an AdaBoost technique with a window size of 128 samples for the TUG test is 92%, which is an improvement of at least 15% compared to that of the typical subtask segmentation approach using machine-learning models only.

## Figures and Tables

**Table 1:** List of the extracted features.

No.	Description
$f_{1}$–$f_{8}$	Mean of $a_{x}$, $a_{y}$, $a_{z}$, $\omega_{x}$, $\omega_{y}$, $\omega_{z}$, $A_{T}$, $G_{T}$
$f_{9}$–$f_{16}$	Standard Deviation of $a_{x}$, $a_{y}$, $a_{z}$, $\omega_{x}$, $\omega_{y}$, $\omega_{z}$, $A_{T}$, $G_{T}$
$f_{17}$–$f_{24}$	Variance of $a_{x}$, $a_{y}$, $a_{z}$, $\omega_{x}$, $\omega_{y}$, $\omega_{z}$, $A_{T}$, $G_{T}$
$f_{25}$–$f_{32}$	Maximum of $a_{x}$, $a_{y}$, $a_{z}$, $\omega_{x}$, $\omega_{y}$, $\omega_{z}$, $A_{T}$, $G_{T}$
$f_{33}$–$f_{40}$	Minimum of $a_{x}$, $a_{y}$, $a_{z}$, $\omega_{x}$, $\omega_{y}$, $\omega_{z}$, $A_{T}$, $G_{T}$
$f_{41}$–$f_{48}$	Range of $a_{x}$, $a_{y}$, $a_{z}$, $\omega_{x}$, $\omega_{y}$, $\omega_{z}$, $A_{T}$, $G_{T}$
$f_{49}$–$f_{56}$	Kurtosis of $a_{x}$, $a_{y}$, $a_{z}$, $\omega_{x}$, $\omega_{y}$, $\omega_{z}$, $A_{T}$, $G_{T}$
$f_{57}$–$f_{64}$	Skewness of $a_{x}$, $a_{y}$, $a_{z}$, $\omega_{x}$, $\omega_{y}$, $\omega_{z}$, $A_{T}$, $G_{T}$

**Table 2:** The preliminarily investigated results of the performances of multi-classifiers and single classifiers.

	SVM classifier with a 96-sample window size	
	Multi-classifier (average of four classifiers)	Single classifier
Sensitivity (%)	88.17	81.30
Precision (%)	88.79	82.72
Accuracy (%)	89.93	81.27
	SVM classifier with a 128-sample window size	
	Multi-classifier (average of four classifiers)	Single classifier
Sensitivity (%)	87.81	82.33
Precision (%)	88.88	83.05
Accuracy (%)	90.53	83.71
	SVM classifier with a 160-sample window size	
	Multi-classifier (average of four classifiers)	Single classifier
Sensitivity (%)	87.36	82.56
Precision (%)	88.89	82.88
Accuracy (%)	90.74	84.34

**Table 3:** The performance results of each phase in subtask identification system with window sizes of 96, 128, and 160 samples.

		Phase												
		Preoperative			Postoperative			Postoperative 2-Week			Postoperative 6-Week			Overall
Window Size	Technique	Acc. (%)	Sen. (%)	Pre. (%)	Acc. (%)	Sen. (%)	Pre. (%)	Acc. (%)	Sen. (%)	Pre. (%)	Acc. (%)	Sen. (%)	Pre. (%)	Acc. (%)
**96**	**SVM**	94.04	91.79	92.41	85.33	85.93	85.99	89.70	87.25	87.60	**90.66**	87.72	89.17	89.93
	**kNN**	91.47	87.25	90.89	80.12	79.46	82.26	87.93	83.74	87.46	89.26	86.62	88.09	87.20
	**NB**	92.48	90.26	90.45	77.62	79.73	77.96	86.86	84.10	84.45	88.23	86.17	85.39	86.30
	DT	92.78	90.37	90.91	72.00	78.33	79.45	**92.01**	88.19	89.73	89.30	87.07	87.07	86.52
	**AdaBoost**	**94.29**	90.62	93.03	91.78	86.28	88.57	87.43	84.20	84.74	89.82	87.12	87.56	90.83
**128**	**SVM**	91.47	86.98	90.98	89.98	88.24	88.19	88.46	83.52	87.80	88.57	85.69	87.05	89.62
	**kNN**	91.47	86.98	90.98	73.42	76.16	78.14	88.46	83.52	87.80	87.85	83.72	86.25	85.30
	**NB**	91.59	89.43	89.48	88.28	83.89	82.78	89.80	85.87	87.44	88.67	86.23	86.02	89.59
	**DT**	92.10	89.24	90.07	74.69	80.09	80.38	91.00	87.12	88.19	89.55	86.33	87.29	86.84
	**AdaBoost**	93.75	91.00	92.29	**93.32**	87.82	91.34	91.04	86.15	88.67	90.44	87.50	88.67	92.14
**160**	**SVM**	92.71	89.29	91.10	89.93	87.83	87.36	90.61	86.45	88.76	89.70	85.88	88.32	90.74
	**kNN**	88.73	82.29	89.54	66.98	69.91	74.88	82.28	75.91	83.01	87.47	84.41	86.62	81.37
	**NB**	90.89	88.23	88.73	89.33	83.91	83.23	90.37	85.82	88.4	89.08	86.27	87.03	89.92
	**DT**	92.21	88.82	90.41	73.56	78.92	78.76	90.13	85.80	87.68	89.16	85.57	86.94	86.27
	**AdaBoost**	92.56	89.18	90.90	92.89	87.26	90.07	91.15	86.20	88.95	89.73	86.20	88.22	91.58

**Table 4:** The performances of each subtask (highest performances of each phase).

	**Using an AdaBoost Technique with a Window Size of 96 Samples in the Preoperative Phase**								
	***Initial Sitting***	***Sit-to-Stand***	***Walking-Out***	***Turning***	***Walking-In***	***Turning Around***	***Stand-to-Sit***	***Ending Sitting***	***Overall***
**Sensitivity (%)**	96.20	84.84	97.81	81.89	98.43	89.07	80.84	95.89	90.62
**Precision (%)**	99.03	90.25	96.20	94.33	94.41	85.67	92.35	92.01	93.03
**Accuracy (%)**	--	--	--	--	--	--	--	--	94.29
	**Using an AdaBoost Technique with a Window Size of 128 Samples in the Postoperative Phase**								
**Sensitivity (%)**	95.35	69.38	98.58	83.61	97.59	88.50	73.59	95.92	87.82
**Precision (%)**	90.30	94.26	95.21	95.19	94.57	84.07	91.51	85.62	91.34
**Accuracy (%)**	--	--	--	--	--	--	--	--	93.32
	**Using a DT Technique with a Window Size of 96 Samples in the Postoperative 2-Week Phase**								
**Sensitivity (%)**	93.97	76.07	97.74	87.42	95.63	87.88	80.36	86.41	88.19
**Precision (%)**	95.13	84.42	94.88	93.37	94.44	73.14	88.33	94.15	89.73
**Accuracy (%)**	--	--	--	--	--	--	--	--	92.01
	**Using an SVM Technique with a Window Size of 96 Samples in the Postoperative 6-Week Phase**								
**Sensitivity (%)**	95.65	82.51	95.08	82.52	92.03	82.32	74.73	96.90	87.72
**Precision (%)**	98.22	89.23	94.80	92.80	90.56	73.44	89.44	84.88	89.17
**Accuracy (%)**	--	--	--	--	--	--	--	--	90.66
